# Multimorbidity patterns and cognitive frailty in adults aged over 50 years: China perspective

**DOI:** 10.3389/fpubh.2025.1701955

**Published:** 2025-11-26

**Authors:** Jiehua Zhu, Chenxi Ren, Tingjun Hu, Jun Jin, Qihao Guo

**Affiliations:** Department of Gerontology, Shanghai Sixth People's Hospital, Shanghai Jiao Tong University School of Medicine, Shanghai, China

**Keywords:** multimorbidity, slow gait, cognitive frailty, aged over 50 years, mild cognitive impairment

## Abstract

**Introduction:**

The multimorbidity underlying in cognitive frailty remain poorly understood.

**Methods:**

A cross-sectional study was performed in non-dementia Chinese aged over 50 years, Cognitive frailty (cognitive impairment and slow gait [SG]). Cardiovascular and cerebrovascular diseases (CCVD) (heart disease, type 2 diabetes, hypertension, hyperlipidemia and cerebral infarction). Continuous variables were evaluated using one-way analysis of variance (ANOVA). Categorical variables were assessed using the chi-square test. Pairwise comparisons were performed using the Bonferroni-corrected column proportion test (z-test). Multiple logistic regression analysis was conducted to identify factors associated with cognitive frailty.

**Results:**

A cross-sectional study included 571 males and 1,182 non-demented Chinese aged ≥50 years, categorized into 6 groups: normal, SG, subjective cognitive decline alone (SCD-A), mild cognitive impairment alone (MCI-A), subjective cognitive decline with slow gait (SCD-SG), and mild cognitive impairment with slow gait (MCI-SG). Cognitive frailty was defined as cognitive impairment plus SG. Walking speed and hand grip strength were lower in SG, SCD-SG, and MCI-SG groups (*p* < 0.05). SCD-SG and MCI-SG groups had higher prevalence of ≥3 chronic conditions (*p* < 0.05); SCD-SG and MCI-SG groups showed higher ≥3 CCVD (9.5, 9.2%; *p* < 0.05) in female. SCD-SG group had higher risk of 1–2 chronic conditions (OR = 1.81, 95%CI = 1.06–3.07) and ≥3 chronic conditions (OR = 3.79, 95%CI = 1.96–7.36).

**Conclusion:**

Cognitive frailty in Chinese aged ≥50 years increases risk of ≥3 chronic diseases, with SCD-SG group at highest risk, highlighting the need for attention to cognitive frailty and multimorbidity.

## Introduction

The aging global population has led to a rising prevalence of aging-related disorders. Alzheimer’s disease (AD), osteoarthritis, and cardiovascular and cerebrovascular diseases (CCVD), including type 2 diabetes, atherosclerosis, and hypertension, are significant age-related conditions (1). While these diseases are closely linked, the association between the pre-AD stage and these chronic disorders remains incompletely understood. Accumulating evidence highlights that cognitive and physical impairments in late life are interrelated through shared pathophysiological mechanisms and may represent a unified complex phenotype (2). Importantly, gait disorders and cognitive decline, which progress with aging, are regarded as primary risk factors for falls in older dementia patients (3, 4). The presence of both slow gait (SG) and objective cognitive impairment was linked to the highest risk of developing dementia and is considered the phenotype of cognitive frailty most susceptible to dementia progression (5).

Slow gait (SG) is recognized as an early symptom of dementia, and its severity worsens with disease progression compared to healthy older adults (6). Subjective cognitive decline (SCD) and mild cognitive impairment (MCI) are early manifestations of AD (7): SCD is characterized by a subjective perception of memory decline or mild objective neurocognitive impairment, while MCI represents a transitional stage between normal aging and mild dementia, with cognitive deficits that do not significantly impact daily functioning. Previous studies have shown that the co-occurrence of MCI and SG is associated with a higher risk of dementia and disability than either condition alone (8, 9). According to the consensus by the International Academy on Nutrition and Aging and the International Association of Gerontology and Geriatrics, cognitive frailty is defined by reduced cognitive reserve and motor decline (10), and research in this field has increasingly focused on motor decline and gait variables due to the critical role of motor function in the interplay between cognitive performance, cognitive impairment, and physical frailty (10). Notably, individual components of frailty have been linked to specific cognitive domains: slow gait is associated with executive function, attention, processing speed (11), word recall, and logical memory (12); weak grip predicts changes in executive function (12); and physical activity correlates with changes in executive function and word recall (12). However, among these factors, only motor performance has had its validity and reliability formally established in previous research (13, 14). Building on these findings, we defined cognitive frailty as the coexistence of cognitive decline and slow gait in the present study (5), a concept that holds significant clinical and research potential for better stratifying the risk of dementia and functional disabilities in older adults (15).

Slow gait serves as a key objective marker of this defined cognitive frailty. Understanding the prevalence of age-related diseases in individuals with cognitive frailty could facilitate early detection and intervention for AD and other age-related conditions, thereby alleviating the substantial burden on healthcare and long-term care systems. Cognitive frailty itself has also been associated with disability and death (16), though the heterogeneity of objective frailty assessment tools has led to inconsistent reliability evaluations of cognitive frailty across studies. Additionally, the limited reliability and inherent variability in the operational metrics of the Cardiovascular Health Study frailty criteria further hinder the establishment of a standardized definition for cognitive frailty. Despite these considerations, few studies have comprehensively explored multimorbidity in cognitive frailty—particularly in the specific subgroups of cognitive decline combined with slow gait, namely SCD plus SG (SCD-SG) and MCI plus SG (MCI-SG).

Therefore, our study aimed to investigate the patterns of multimorbidity in cognitive frailty that consists of cognitive decline and slow gait among Chinese non-dementia adults aged over 50 years. A major strength of this article lies in the combination of cognitive function severity levels and gait speed.

## Methods

### Study population

Between January 2019 and June 2023, 3,034 participants were enrolled from the Department of Geriatrics at Shanghai Sixth People’s Hospital. Trained staff conducted neuropsychological tests for all participants in a standardized neuropsychological assessment room. Exclusion criteria included: (1) age under 50 years (*n* = 303); (2) diagnosis of dementia based on the American Psychiatric Association’s Diagnostic and Statistical Manual of Mental Disorders, 4th edition (DSM-IV) (*n* = 645); (3) a history of psychological disorders (*n* = 32); and (4) individuals with physical disabilities who were unable to provide gait speed measurements or unavailable gait speed records (*n* = 301). After applying these criteria, 1,753 participants were included in the final analysis. The study protocol was approved by the Ethics Committee of Shanghai Sixth People’s Hospital (approval number: 2019–041), and all participants provided written informed consent in compliance with the Helsinki Declaration.

### Measurements at baseline

Patients’ demographic characteristics, chronic diseases, medical history, and lifestyle factors—including age, sex, smoking and drinking status, heart disease, hypertension, type 2 diabetes, osteoporosis, cerebral infarction, fracture, and cirrhosis—were verified through medical record reviews by experienced clinicians. Heart disease included a history of coronary artery disease and arrhythmia, while chronic lung disease encompassed chronic obstructive pulmonary disease and chronic bronchitis. Cerebral infarction was diagnosed based on a history of ischemic events confirmed by brain CT or MRI scans. Body mass index (BMI, kg/m^2^) was calculated as weight (kg) divided by height squared (m^2^). Hand grip strength (kg) was assessed using a dynamometer (WCS-100, Nantong, China).

The SCD group included individuals who reported subjective cognitive decline without objective neuropsychological test abnormalities, as well as those with detectable cognitive changes using sensitive neuropsychological measures during the preclinical stage of AD. MCI inclusion criteria were adapted from Petersen RC’s guidelines: (1) cognitive complaints reported by the participant, informant, nurse, or physician within the past year; (2) normal cognitive function (Montreal Cognitive Assessment Basic Edition [MoCA-BC] scores: primary school 19–14, secondary school 22–16, university and above 24–17); (3) objective cognitive impairment, defined as performance at least 1.5 SD below age- and education-adjusted norms on standardized neuropsychological tests; (4) minimal impairment in daily living activities, with no more than one item significantly affected on the Chinese version of the Activities of Daily Living Scale (ADLs) or a total score < 26 (17); and (5) no diagnosis of dementia (18, 19). Gait speed was assessed using the timed up-and-go test over a 6-meter distance. The threshold for gait speed varies in the literature. Merchant RA et al. defined SG as <1 m/s (20), while other studies proposed thresholds of <0.6 m/s (21, 22) or <0.8 m/s (23) to identify individuals at higher risk of poor health outcomes. In our study, to ensure a rigorous analysis across age groups, we first focused on participants aged 50–59 years and defined SG as a gait speed below 0.6 m/s.

The participants in this study were divided into six groups: the normal group, the SG group, the subjective cognitive decline alone (SCD-A) group, the mild cognitive impairment alone (MCI-A) group, the group with concurrent SCD and SG (SCD-SG), and the group with concurrent MCI and SG (MCI-SG) group. The “cognitive frailty groups” are characterized by concurrent cognitive decline and SG, corresponding specifically to the SCD-SG group and the MCI-SG group.

### Statistical analysis

Statistical analyses were carried out using SAS version 9.4 (SAS Institute, Cary, NC). Continuous variables, such as age, education level, hand grip strength, gait speed, and neuropsychological test scores, were reported as mean ± standard deviation (SD). Differences among groups for continuous variables were evaluated using one-way analysis of variance (ANOVA). For categorical variables, including gender, smoking status, alcohol consumption status, and the prevalence of chronic diseases, group differences were assessed using the chi-square test. Pairwise comparisons were performed using the Bonferroni-corrected column proportion test (z-test). Multiple logistic regression analysis was conducted to identify factors associated with MCI-SG and SCD-SG. A *p*-value < 0.05 was considered statistically significant. Figures were prepared using GraphPad Prism version 9.

## Results

### Demographic and clinical characteristics

A total of 1,753 participants (571 males and 1,182 females) were divided into six groups: normal, SG, SCD-A, MCI-A, SCD-SG, and MCI-SG. Each group was further stratified by gender to explore potential gender-specific differences in demographic and clinical characteristics.

### Gender and group distribution

Females accounted for 67.4% of the total participants, with no significant difference in gender distribution across the six groups (*p* = 0.855). The proportion of participants in each group was as follows: normal group (34.5%), SG group (10.0%), SCD-A group (17.7%), MCI-A group (16.8%), SCD-SG group (8.3%), and MCI-SG group (12.7%).

### Age

In the female subgroup, participants in the SG (68.4 ± 7.8 years), SCD-SG (67.1 ± 8.1 years), and MCI-SG (68.4 ± 7.3 years) groups were significantly older than those in the normal, SCD-A, and MCI-A groups (*p* < 0.05). A similar age trend was observed in the male subgroup, with the SG (69.4 ± 7.7 years), SCD-SG (69.1 ± 6.1 years), and MCI-SG (69.7 ± 7.2 years) groups being significantly older than the other three groups (*p* < 0.05).

### Lifestyle and anthropometric indicators

Smoking status, alcohol consumption, and waist circumference showed no significant differences across the six groups, regardless of gender. Regarding BMI, the SG group had a higher BMI in males, while the SCD-SG (24.3 ± 3.8 kg/m^2^) and MCI-SG (24.2 ± 3.5 kg/m^2^) groups had higher BMI in females (*p* < 0.05).

### Physical function

Regardless of gender, walking speed was significantly slower in the SG (males: 0.51 ± 0.09 m/s; females: 0.49 ± 0.09 m/s), SCD-SG (males: 0.51 ± 0.06 m/s; females: 0.50 ± 0.08 m/s), and MCI-SG (males: 0.50 ± 0.09 m/s; females: 0.48 ± 0.08 m/s) groups compared to the normal, SCD-A, and MCI-A groups (*p* < 0.05). A similar trend was observed for hand grip strength across the six groups (*p* < 0.05).

### Education level and cognitive function

The MCI-SG (males: 11.3 ± 3.7 years; females: 10.4 ± 3.0 years) and MCI-A (males: 11.6 ± 2.9 years; females: 10.6 ± 3.5 years) groups had significantly lower years of education than the other four groups (*p* < 0.05). For cognitive function (assessed using the MoCA-BC and Chinese version of Addenbrooke’s Cognitive Examination-III (ACE-III-CV) total scores), the MCI-SG and MCI-A groups had the lowest scores, followed by the SCD-SG group (all *p* < 0.05).

### APOEε4 carrier status and gender-related differences

There was no significant difference in APOEε4 carrier status between males and females, indicating that gender may not affect APOEε4 carrier status and thus eliminating potential sample interference from higher cognitive risk associated with more APOEε4 carriers in either gender. Additionally, some groups showed significant gender differences in age, smoking status, alcohol consumption, years of education, BMI, waist circumference, hand grip strength, and MoCA-BC scores.

All demographic and clinical characteristic data are summarized in Table 1.

**Table 1:** Baseline characteristics of participants in six groups.

Characteristics	Normal (*n* = 605)	SG (*n* = 176)	SCD-A (*n* = 310)	MCI-A (*n* = 294)	SCD-SG (*n* = 146)	MCI-SG (*n* = 222)	*p*
Sex *n* (%)	203 (33.6)	61 (34.7)	91 (32.3)	95 (32.3)	41 (28.1)	80 (36.0)	0.855
Age (years) (*n* = 1,753)							
Male (*n* = 571)	66.2 ± 7.1^bef*^	69.4 ± 7.7^ac^	65.4 ± 7.5^bef^	67.2 ± 6.0^f*^	69.1 ± 6.1^ac^	69.7 ± 7.2^acd^	<0.001
Female (*n* = 1,182)	63.1 ± 6.8^def^	68.4 ± 7.8^acd^	64.0 ± 6.9^bdef^	65.4 ± 6.5^abcef^	67.1 ± 8.1^acd^	68.4 ± 7.3^acd^	<0.001
Smoking *n* (%) (*n* = 930)							
Male (*n* = 391)	71 (50.4)*	17 (43.6)*	25 (41.0)*	40 (58.0)*	14 (50.0)*	28 (50.9)*	0.596
Female (*n* = 539)	3 (1.65)	0 (0)	1 (1.11)	3 (3.00)	0 (0)	2 (2.86)	0.576
Drinking *n* (%) (*n* = 1,674)							
Male (*n* = 543)	74 (39.4)*	23 (39.0)*	35 (40.7)*	38 (41.8)*	18 (45.0)*	29 (36.3)*	0.970
Female (*n* = 1,131)	27 (7.01)	5 (4.55)	17 (8.21)	12 (6.38)	7 (6.80)	10 (7.25)	0.877
Education (years) (*n* = 1,741)							
Male (*n* = 565)	12.9 ± 3.2^df*^	12.9 ± 3.2^df^	12.6 ± 3.5^f*^	11.6 ± 2.9^abe*^	12.9 ± 4.0^df*^	11.3 ± 3.7^abce^	<0.001
Female (*n* = 1,176)	12.2 ± 3.0^cdef^	12.2 ± 3.3^def^	11.6 ± 3.1^acf^	10.6 ± 3.5^abc^	11.3 ± 3.0^abf^	10.4 ± 3.0^abcf^	<0.001
BMI (kg/m^2^) (*n* = 1,649)							
Male (*n* = 531)	24.4 ± 2.9^c*^	25.2 ± 3.1^c*^	23.6 ± 3.1^abf^	23.6 ± 3.1^abf^	24.2 ± 2.1	24.6 ± 4.5*	0.020
Female (*n* = 1,118)	23.1 ± 2.9^ef^	23.4 ± 3.7*	23.1 ± 3.4^ef^	23.1 ± 2.8^ef^	24.3 ± 3.8^abcd^	24.2 ± 3.5^ace^	<0.001
Waist circumference (cm) (*n* = 1,288)							
Male (*n* = 416)	89.1 ± 12.4*	91.2 ± 9.7*	86.9 ± 12.5*	88.1 ± 12.5*	90.9 ± 8.9*	90.2 ± 11.8*	0.237
Female (*n* = 872)	82.9 ± 12.2	82.5 ± 10.8	81.9 ± 10.5	81.6 ± 14.2	84.4 ± 11.9	85.1 ± 12.1	0.163
Gait speed (m/s) (*n* = 1,735)							
Male (*n* = 571)	0.74 ± 0.11^bdef^	0.51 ± 0.09^acd^	0.74 ± 0.11^bdef^	0.71 ± 0.09^abde^	0.51 ± 0.06^acd^	0.5 ± 0.09^acd^	<0.001
Female (*n* = 1,182)	0.75 ± 0.13^bdef^	0.49 ± 0.09^acd^	0.74 ± 0.11^bef^	0.72 ± 0.11^abef^	0.50 ± 0.08^acd^	0.48 ± 0.08^acd^	<0.001
Hand grip strength (kg) (*n* = 1,735)							
Male (*n* = 567)	32.1 ± 7.7^bef*^	28.9 ± 7.8^acf*^	31.2 ± 7.6^bef*^	30.5 ± 7.4^f*^	28.8 ± 6.5^acf*^	25.6 ± 8.3^abcde*^	<0.001
Female (*n* = 1,166)	21.0 ± 4.6^bded^	19.6 ± 6.0^ac^	21.1 ± 9.4^bdef^	19.7 ± 4.2^acf^	18.4 ± 5.0^ac^	18.1 ± 5.2^ace^	<0.001
MoCA-BC total scores (*n* = 1,750)							
Male (*n* = 570)	25.6 ± 2.5^cdef^	25.1 ± 3.2^cdef^	23.8 ± 3.4^abdf^	21.7 ± 2.9^abcef^	23.4 ± 2.9^abdf^	20.1 ± 3.6^abcde^	<0.001
Female (*n* = 1,180)	25.7 ± 2.8^bcdef^	24.6 ± 3.0^acdef^	24.3 ± 3.6^abef^	21.8 ± 3.7^abcef^	23.2 ± 3.9^abcdf^	20.2 ± 3.9^abcde^	<0.001
ACE-III-CV total scores (*n* = 908)							
Male (*n* = 307)	84.9 ± 6.9^cdef*^	83.7 ± 6.4^cdef*^	78.2 ± 8.0^abcf^	75.1 ± 8.0^abcef*^	79.3 ± 6.7^abdf^	70.8 ± 8.6^abcde*^	<0.001
Female (*n* = 601)	81.3 ± 7.2^cdef^	78.9 ± 7.7^df^	76.5 ± 9.7^adf^	70.3 ± 9.6^abce^	75.7 ± 9.0^adf^	67.6 ± 11.0^abce^	<0.001
APOE ε4 carrier (*n* = 654)							
Male (*n* = 233)	19 (23.5)^*^	5 (19.2)	5 (13.5)	11 (28.2)	3 (18.8)	12 (35.3)	0.248
Female (*n* = 421)	21 (15.7)	6 (12.8)	15 (18.1)	14 (24.1)	8 (18.2)	18 (32.7)	0.105

SG, slow gait; SCD-A, subjective cognitive decline alone; MCI-A, mild cognitive impairment alone; SCD-SG, SCD plus slow gait; MCI-SG, MCI plus slow gait; BMI, Body mass index; MoCA-BC, Montreal Cognitive Assessment Basic Edition; ACE-III-CV, Chinese version of Addenbrooke’s Cognitive Examination-III. ^a^Significantly different from the normal group. ^b^Significantly different from the SG group. ^c^Significantly different from the SCD-A group. ^d^Significantly different from the MCI-A group. ^e^Significantly different from the SCD-SG group. ^f^Significantly different from the MCI-SG group. ^*^Significantly different between male and female.

### The difference prevalence of chronic conditions and CCVD among normal, SG, SCD-A, MCI-A, SCD-SG and MCI-SG groups

**Figure 1:**
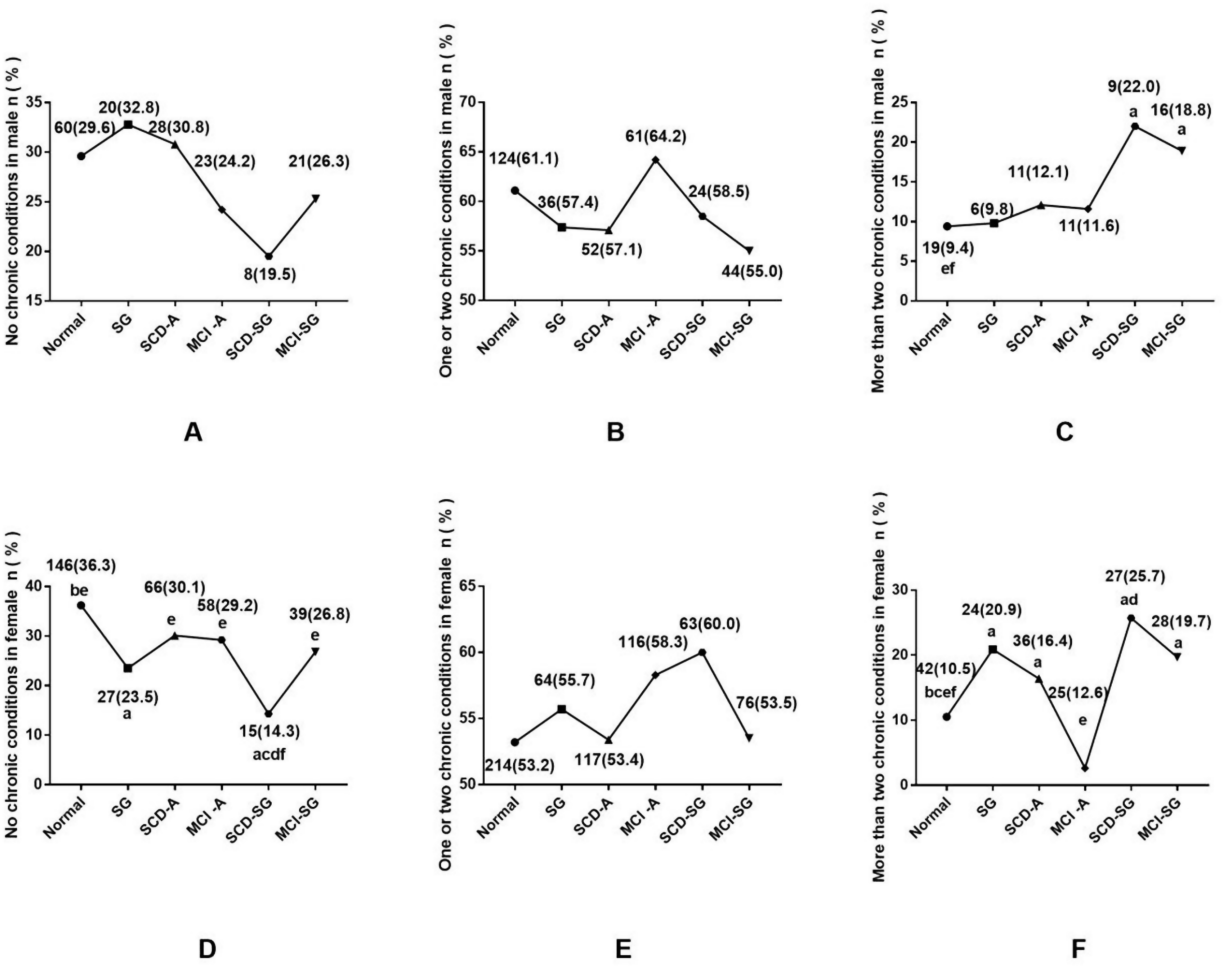
**(A)** The difference prevalence of no chronic conditions among six groups in male. **(B)** The difference prevalence of one or two chronic conditions among six groups in male. **(C)** The difference prevalence of more than two chronic conditions among six groups in male. **(D)** The difference prevalence of no chronic conditions among six groups in female. **(E)** The difference prevalence of one or two chronic conditions among six groups in female. **(F)** The difference prevalence of more than two chronic conditions among six groups in female. ^a^Significantly different from the normal group. ^b^Significantly different from the SG group. ^c^Significantly different from the SCD-A group. ^d^Significantly different from the MCI-A group. ^e^Significantly different from the SCD-SG group. ^f^Significantly different from the MCI-SG group.

Figure 1 displayed the prevalence of chronic conditions, categorized by the number of conditions (none, one or two, and more than two) across six groups. In males, the SCD-SG group (22.0%) and MCI-SG group (18.8%) had a significantly higher prevalence of more than two chronic conditions compared to the normal group (*p* < 0.05). No significant differences were observed for none or one to two chronic conditions. Among females, the proportion of participants with no chronic conditions was highest in the normal group and lowest in the SCD-SG group (36.3% vs. 14.3%, *p* < 0.05). In contrast, the prevalence of more than two chronic conditions was significantly higher in the SCD-SG group (25.7%) and MCI-SG group (19.7%) compared to the normal group (10.5%, *p* < 0.05).

**Figure 2:**
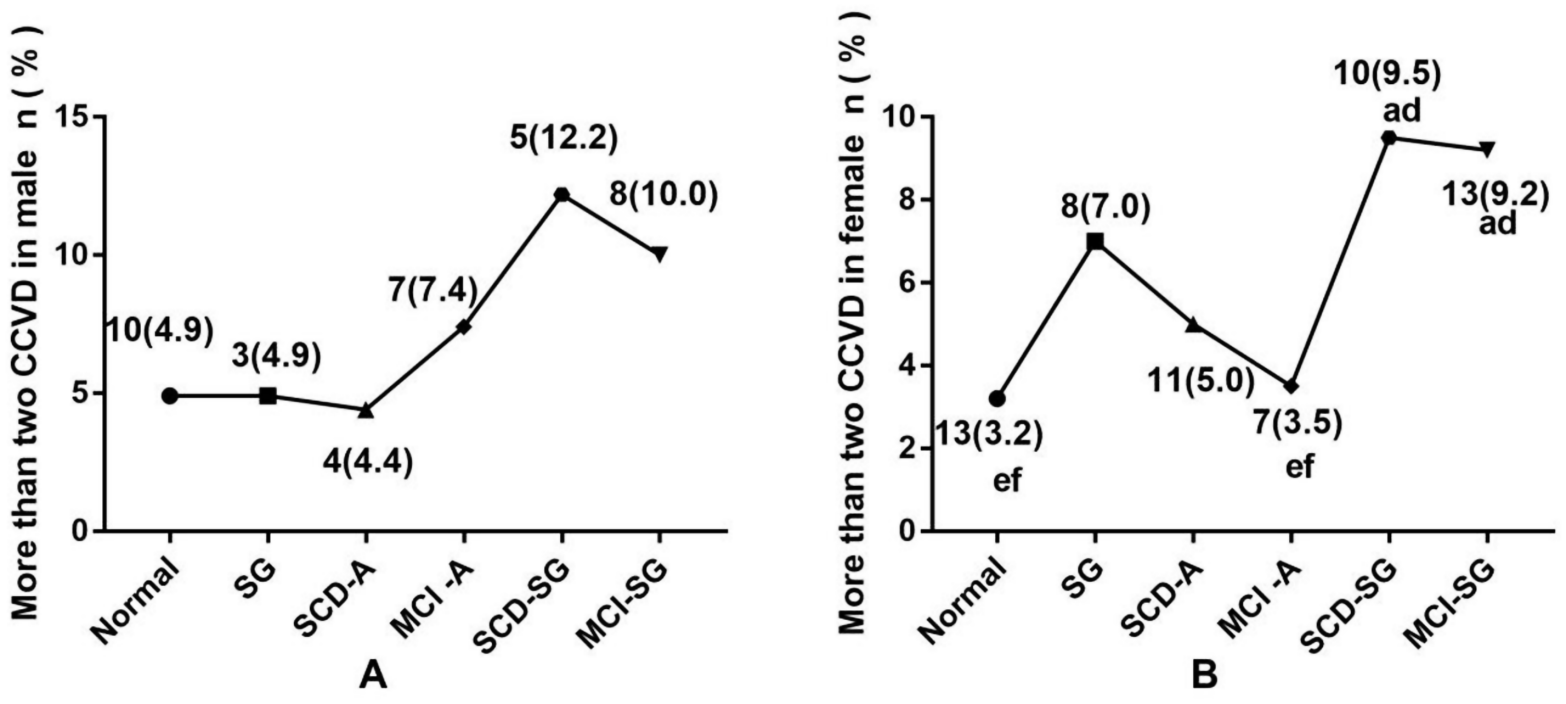
**(A)** The difference prevalence of more than two CCVD among six groups in male. **(B)** The difference prevalence of more than two CCVD among six groups in female. CCVD, Cardiovascular and cerebrovascular diseases. ^a^Significantly different from the normal group. ^b^Significantly different from the SG group. ^c^Significantly different from the SCD-A group. ^d^Significantly different from the MCI-A group. ^e^Significantly different from the SCD-SG group. ^f^Significantly different from the MCI-SG group.

Cardiovascular and cerebrovascular diseases (CCVD), encompassing heart disease, type 2 diabetes, hypertension, hyperlipidemia, and cerebral infarction, were also analyzed. The prevalence of more than two CCVD was significantly higher in the SCD-SG group (9.5%) and MCI-SG group (9.2%) among females (*p* < 0.05). A similar trend was observed in males, though the differences were not statistically significant (Figure 2).

### Chronic conditions among normal, SG, SCD-A, MCI-A, SCD-SG and MCI-SG groups

We demonstrated that the prevalence of osteoporosis, cerebral infarction, and fracture varied significantly among the six male subgroups (*p* < 0.05). Osteoporosis prevalence was elevated in the SCD-SG and MCI-SG groups, while cerebral infarction and fracture rates were highest in the MCI-SG group. In females, significant differences were observed in the prevalence of heart disease, type 2 diabetes, osteoporosis, fracture, liver cirrhosis, and cervical vascular disease across groups (*p* < 0.05), with the SCD-SG and MCI-SG groups showing the highest prevalence of these conditions (Table 2).

**Table 2:** Chronic conditions in six groups.

Diseases	Normal (*n* = 605)	SG (*n* = 176)	SCD-A (*n* = 310)	MCI-A (*n* = 294)	SCD-SG (*n* = 146)	MCI-SG (*n* = 222)	*p*
Heart disease *n* (%) (*n* = 1,753)							
Male (*n* = 571)	14 (6.90)	6 (9.84)	12 (13.2)	6 (6.32)	5 (12.2)	9 (11.3)	0.274
Female (*n* = 1,182)	18 (4.48)^bdef^	13 (11.3)^a^	24 (11.0)	20 (10.1)^a^	14 (13.3)^a^	18 (12.7)^a^	<0.001
Type 2 diabetes *n* (%) (*n* = 1753)							
Male (*n* = 571)	40 (19.7)	8 (13.1)	15 (16.5)	13 (13.7)	9 (22.0)	17 (21.3)	0.859
Female (*n* = 1,182)	34 (8.46)^ef^	14 (12.2)	32 (14.6)	24 (12.1)	20 (19.1)^a^	21 (14.8)^a^	0.005
Hypertension *n* (%) (*n* = 1753)							
Male (*n* = 571)	88 (43.4)^*^	27 (44.3)^*^	37 (40.7)	46 (48.4)^*^	23 (56.1)^*^	36 (43.8)	0.443
Female (*n* = 1,182)	112 (27.9)	40 (34.8)	66 (30.1)	68 (34.2)	39 (37.1)	50 (35.2)	0.360
Hyperlipidemia *n* (%) (*n* = 1753)							
Male (*n* = 571)	37 (18.2)	9 (14.8)	13 (14.3)	23 (24.2)	10 (24.4)	14 (17.5)	0.500
Female (*n* = 1,182)	77 (19.2)	28 (24.3)	50 (22.8)	41 (20.6)	25 (23.8)	24 (16.9)	0.948
Chronic lung disease *n* (%) (*n* = 1753)							
Male (*n* = 571)	15 (7.39)	3 (4.92)	7 (7.69)	9 (9.47)^*^	4 (9.76)	8 (10.00)	0.304
Female (*n* = 1,182)	27 (6.72)	7 (6.09)	10 (4.57)	7 (3.52)	3 (2.86)	11 (7.75)	0.449
Tumour *n* (%) (*n* = 1753)							
Male (*n* = 571)	7 (3.45)	1 (1.64)	2 (2.20)	4 (4.21)	1 (2.44)	3 (3.75)	0.834
Female (*n* = 1,182)	25 (6.22)	7 (6.09)	12 (5.48)	6 (3.02)	7 (6.67)	8 (5.63)	0.540
Osteoporosis *n* (%) (*n* = 1753)							
Male (*n* = 571)	3 (1.48)^d*^	0 (0)^*^	2 (2.20)^d*^	2 (2.11)^ae*^	6 (14.6)^cd^	4 (5.00)^*^	0.003
Female (*n* = 1,182)	45 (11.2)^e^	20 (17.4)	29 (13.2)^e^	32 (16.1)^e^	26 (24.8)^acd^	21 (14.8)	0.020
Cerebral infarction *n* (%) (*n* = 1753)							
Male (*n* = 571)	6 (2.96)^b^	5 (8.20)^af^	1 (1.10)^d^	8 (8.42)^c*^	1 (2.44)	8 (10.0)^b^	0.039
Female (*n* = 1,182)	15 (3.73)	7 (6.09)	10 (4.57)	5 (2.51)	4 (3.81)	11 (7.75)	0.303
Fracture *n* (%) (*n* = 1753)							
Male (*n* = 571)	8 (3.94)^cf*^	3 (4.92)^*^	3 (3.30)^a*^	8 (8.42)	4 (9.76)	7 (8.75)^a^	0.038
Female (*n* = 1,182)	32 (7.96)^bdef^	20 (17.4)^a^	30 (13.7)	27 (13.6)^a^	19 (18.1)^a^	22 (15.5)^a^	0.004
Liver disease *n* (%) (*n* = 1753)							
Male (*n* = 571)	6 (2.96)	1 (1.64)	4 (4.40)	0 (0)	0 (0)	7 (8.75)	0.203
Female (*n* = 1,182)	5 (1.24)^ef^	1 (0.87)^e^	7 (3.20)^e^	4 (2.01)^e^	9 (8.57)^abcd^	7 (4.93)^a^	<0.001
Hypothyroidism *n* (%) (*n* = 1753)							
Male (*n* = 571)	1 (0.49)^*^	1 (1.64)	1 (1.10)^*^	0 (0)	1 (2.44)	0 (0)	0.856
Female (*n* = 1,182)	22 (5.47)	6 (5.22)	18 (8.22)	7 (3.52)	12 (11.4)	4 (2.82)	0.882
Hyperthyroidism *n* (%) (*n* = 1753)							
Male (*n* = 571)	2 (0.99)	0 (0)	1 (1.10)	0 (0)	0 (0)	0 (0)	0.230
Female (*n* = 1,182)	8 (1.99)	2 (1.74)	8 (3.65)	4 (2.01)	0 (0)	4 (2.82)	0.986
Lowervascular *n* (%) (*n* = 1753)							
Male (*n* = 571)	0 (0)	0 (0)	1 (1.01)	1 (1.05)	0 (0)	1 (1.25)	0.171
Female (*n* = 1,182)	4 (1.00)	1 (0.87)	2 (0.91)	4 (2.01)	2 (1.90)	2 (1.41)	0.368
Cervicalvascular *n* (%) (*n* = 1753)							
Male (*n* = 571)	4 (1.97)	0 (0)	3 (3.30)	4 (4.21)	0 (0)	3 (3.75)	0.349
Female (*n* = 1,182)	5 (1.24)^e^	1 (0.87)	7 (3.20)	4 (2.01)	5 (4.76)^a^	5 (3.52)	0.027
Chronic infection *n* (%) (*n* = 1753)							
Male (*n* = 571)	3 (1.48)	0 (0)	3 (3.30)^*^	0 (0)	0 (0)	2 (2.50)	0.890
Female (*n* = 1,182)	5 (1.24)	2 (1.74)	0 (0)	3 (1.51)	2 (1.90)	4 (2.82)	0.242

SG, slow gait; SCD-A, subjective cognitive decline alone; MCI-A, mild cognitive impairment alone; SCD-SG, SCD plus slow gait; MCI-SG, MCI plus SG. ^a^Significantly different from the normal group. ^b^Significantly different from the SG group. ^c^Significantly different from the SCD-A group. ^d^Significantly different from the MCI-A group. ^e^Significantly different from the SCD-SG group. ^f^Significantly different from the MCI-SG group. *Significantly different between male and female.

### Odd ratios (ORs) and 95% confidence intervals (CIs) for the associations of the number of chronic conditions with risk of SG, SCD-A, MCI-A, SCD-SG and MCI-SG

In all participants, when adjusted for age, gender, education, BMI, HGS, and MoCA-BC, compared to the normal group, it was found that the SCD-A group had a greater risk of developing at least two chronic conditions (OR = 1.89, 95% CI: 1.14–3.12), whereas the SCD-SG group had a 2.79 times higher risk of developing more than two chronic conditions (Table 3).

**Table 3:** Odd ratios (ORs) and 95% confidence intervals (CIs) for the associations of the number of chronic conditions with risk of SG, SCD-A, MCI-A, SCD-SG and MCI-SG.

Number of chronic conditions	Unadjusted OR (95%CI)	Model 1 OR (95%CI)	Model 2 OR (95%CI)
SG			
0	1 (reference)	1 (reference)	1 (reference)
1–2	1.28 (0.87–1.89)	1.01 (0.68–1.51)	0.94 (0.62–1.45)
> = 3	**2.16 (1.26–3.70)**	1.43 (0.81–2.51)	1.33 (0.73–2.44)
SCD-A			
0	1 (reference)	1 (reference)	1 (reference)
1–2	1.10 (0.81–1.49)	1.08 (0.79–1.48)	1.06 (0.76–1.47)
> = 3	**1.69 (1.08–2.65)**	**1.67 (1.04–2.66)**	**1.89 (1.14–3.12)**
MCI-A			
0	1 (reference)	1 (reference)	1 (reference)
1–2	1.33 (0.95–1.83)	1.14 (0.82–1.58)	1.11 (0.77–1.59)
> = 3	1.50 (0.92–2.44)	1.26 (0.76–2.09)	1.43 (0.82–2.49)
SCD-SG			
0	1 (reference)	1 (reference)	1 (reference)
1–2	**2.31 (1.41–3.77)**	**1.89 (1.15–3.12)**	**1.81 (1.06–3.07)**
> = 3	**5.29 (2.91–9.59)**	**3.89 (2.10–7.19)**	**3.79 (1.96–7.36)**
MCI-SG			
0	1 (reference)	1 (reference)	1 (reference)
1–2	1.24 (0.87–1.77)	0.88 (0.60–1.28)	0.81 (0.53–1.26)
> = 3	**2.46 (1.51–4.00)**	1.58 (0.95–2.64)	1.55 (0.84–2.77)

Model 1: adjusted for age, sex and education. Model 2: adjusted for age, sex, education, BMI, HGS and MoCA-BC. Bold formatting signifies statistical significance.

In males, it was found that the SCD-A group had a greater risk of developing more than two chronic conditions (OR = 4.76, 95% CI: 1.44–15.78) (Table 4).

**Table 4:** Odd ratios (ORs) and 95% confidence intervals (CIs) for the associations of the number of chronic conditions with risk of SG, SCD-A, MCI-A, SCD-SG and MCI-SG in males and females.

Number of chronic conditions	Unadjusted OR (95%CI)	Model 1 OR (95%CI)	Model 2 OR (95%CI)
SG (males)			
0	1 (reference)	1 (reference)	1 (reference)
1–2	0.85 (0.45–1.59)	0.76 (0.40–1.47)	0.80 (0.39–1.61)
> = 3	0.95 (0.33–2.70)	0.97 (0.33–2.83)	0.97 (0.30–3.21)
SG (females)			
0	1 (reference)	1 (reference)	1 (reference)
1–2	1.61 (0.98–2.64)	1.15 (0.69–1.93)	0.98 (0.57–1.68)
> = 3	**3.17 (1.65–6.06)**	1.61 (0.81–3.23)	1.37 (0.65–2.86)
SCD-A (males)			
0	1 (reference)	1 (reference)	1 (reference)
1–2	0.90 (0.52–1.56)	0.91 (0.52–1.59)	0.92 (0.50–1.69)
> = 3	1.24 (0.52–2.95)	1.31 (0.55–3.16)	1.68 (0.62–4.51)
SCD-A (females)			
0	1 (reference)	1 (reference)	1 (reference)
1–2	1.20 (0.83–1.74)	1.16 (0.79–1.69)	1.12 (0.75–1.67)
> = 3	**1.94 (1.14–3.31)**	**1.82 (1.04–3.21)**	**1.99 (1.10–3.62)**
MCI-A (males)			
0	1 (reference)	1 (reference)	1 (reference)
1–2	1.28 (0.73–2.27)	1.20 (0.67–2.16)	1.33 (0.68–2.60)
> = 3	1.51 (0.62–3.66)	1.51 (0.61–3.73)	2.03 (0.70–5.84)
MCI-A (females)			
0	1 (reference)	1 (reference)	1 (reference)
1–2	1.36 (0.93–1.98)	1.10 (0.74–1.64)	0.99 (0.64–1.54)
> = 3	1.54 (0.85–2.75)	1.81 (0.64–2.18)	1.20 (0.62–2.33)
SCD-SG (males)			
0	1 (reference)	1 (reference)	1 (reference)
1–2	1.45 (0.62–3.42)	1.33 (0.56–3.15)	1.38 (0.54–3.53)
> = 3	**3.55 (1.20–10.50)**	**3.48 (1.16–10.41)**	**4.76 (1.44–15.78)**
SCD-SG (females)			
0	1 (reference)	1 (reference)	1 (reference)
1–2	**2.85 (1.56–5.21)**	**2.23 (1.20–4.12)**	**2.00 (1.05–3.82)**
> = 3	**6.41 (3.12–13.17)**	**4.34 (2.04–9.23)**	**3.77 (1.68–8.46)**
MCI-SG (males)			
0	1 (reference)	1 (reference)	1 (reference)
1–2	1.01 (0.55–1.86)	0.85 (0.42–1.58)	0.87 (0.42–1.82)
> = 3	2.26 (0.97–5.22)	2.03 (0.85–4.86)	1.96 (0.66–5.83)
MCI-SG (females)			
0	1 (reference)	1 (reference)	1 (reference)
1–2	1.36 (0.87–2.11)	0.88 (0.55–1.41)	0.77 (0.45–1.33)
> = 3	**2.62 (1.44–4.77)**	1.38 (0.73–2.64)	1.27 (0.61–2.65)

Model 1: adjusted for age and education. Model 2: adjusted for age, education, BMI, HGS and MoCA-BC. Bold formatting signifies statistical significance.

In females,it was found that the SCD-A group had a greater risk of developing more than two chronic conditions (OR = 1.99, 95% CI: 1.10–3.62); the SCD-SG group had a 1.00 time higher risk of developing one or two chronic conditions, 2.77 times higher risk of developing more than two chronic conditions (Table 4).

## Discussion

Over 20% of the older adults in our study exhibited cognitive frailty, characterized by the coexistence of cognitive decline and SG, along with multiple chronic conditions. Notably, Chinese adults aged over 50 years with subjective cognitive decline (SCD) accompanied by SG were more likely to have more than two chronic conditions. This study is the first to systematically investigate the relationship between cognitive frailty, SG, and multimorbidity in non-dementia Chinese adults aged over 50 years.

Cognitive frailty is considered a reversible condition (2), making it an ideal target for preventing asymptomatic preclinical cognitive impairment. We divided cognitive frailty into SCD-SG and mild MCI-SG and revealed differences in chronic disease burden among these phenotypes. We found that individuals with SCD-SG had a significantly higher risk of developing two or more chronic conditions whether in males or females. Growing evidence suggests that interventions, particularly physical activity, are effective in reducing cognitive decline and preventing negative health outcomes (10).

Our study focused on multimorbidity in cognitive frailty, which is the integration of cognitive function severity and gait speed, enabling comprehensive characterization of cognitive frailty. We observed osteoporosis was more prevalent in both genders with concurrent cognitive decline and slow gait (SG), with females at higher risk. Certain age-related metabolic diseases were also more common in women, potentially driven by genetic and hormonal factors. These findings align with prior research reporting elevated burdens of age-related conditions, such as type 2 diabetes, cerebral infarction, heart disease, fractures in individuals with SCD and SG (24, 25). Notably, we detail individual comorbidities instead of relying on aggregate indices like the Charlson Comorbidity Index. This granular approach revealed hypertension, hyperlipidemia, chronic lung disease, tumors, thyroid disorders, and chronic infections were not highly prevalent in our cognitive frailty cohorts—differing from studies that found no significant differences in common metabolic or endocrine conditions across SCD, MCI, and AD groups (26–28). These observations support cognitive frailty’s potential reversibility in preclinical stages: modifiable comorbidities, such as osteoporosis, age-related metabolic diseases identified here are actionable intervention targets. Targeting osteoporosis via lifestyle or pharmacological strategies, for instance, may mitigate cognitive frailty progression, supporting early intervention feasibility. Alignment with prior evidence (24, 25) reinforces addressing multimorbidity in early cognitive frailty, while our distinct profile underscores the need for larger longitudinal studies to refine intervention frameworks.

The association between CCVD and SG, SCD, or cognitive frailty is well supported by prior research, but our study advances this understanding by focusing on their cooccurrence in SCD-SG, MCI-SG. Notably, while the rising prevalence and mortality of cardiovascular disease (CVD) in China underscore the clinical relevance of these links (29), prior work—including a recent study (37) has primarily focused on aggregate multimorbidity burden or isolated cognitive or gait outcomes rather than their combined presentation in preclinical stages.

Some studies reported that cardiovascular and metabolic multimorbidity correlates with incident cognitive frailty in older adults, emphasizing shared inflammatory pathways (38, 39). Our findings align with this mechanistic framework: elevated IL-6 and C-reactive protein have been linked to SG (30), reduced gray matter and hippocampal volume (31, 32)—structures critical for cognition and gait regulation—and subsequent cognitive decline (33). However, we extend this work by demonstrating that these inflammatory pathways also intersect with gender-specific comorbidities in preclinical AD subgroups, a gap not addressed in prior research.

A key novel finding of our study is the higher incidence of liver cirrhosis in females with coexisting cognitive decline and SG—an association rarely reported in cognitive-gait comorbidity research. This may stem from gender-specific metabolic alterations, hormonal fluctuations, malabsorption, or age-related muscle strength decline (34), all of which interact with inflammatory pathways highlighted in the Experimental Gerontology study. Additionally, our observation of poor handgrip strength (a core frailty marker) in SCD-SG and MCI-SG subgroups reinforces prior links between IL-6, reduced muscle mass, and cognitive impairment (35, 36), but contextualizes this within preclinical AD—where intervention potential is greatest. Together, these findings refine the multimorbidity profile of cognitive decline and SG, distinguishing our work from prior aggregate analyses and highlighting novel targets for early intervention. Larger longitudinal studies are needed to confirm these gender-specific associations and explore causal relationships.

However, the present study has several limitations that should be acknowledged. First, data on certain chronic conditions—such as fatty liver disease—were not comprehensively collected. This may have led to underrepresentation of the true burden of multimorbidity in the study population, potentially introducing bias into the analysis of associations between chronic diseases and cognitive outcomes. Second, the relatively small sample size of male participants constrained the statistical power of gender-stratified analyses. As a result, we may have been unable to detect subtle associations between chronic conditions and cognitive decline that are specific to males, limiting the generalizability of our gender-specific findings. Finally, the cross-sectional nature of the study design precluded us from establishing directional or causal relationships between chronic diseases and cognitive decline. It remains unclear whether chronic conditions precede and contribute to cognitive decline, or if early cognitive impairment increases the risk of developing chronic diseases, or if both are driven by shared underlying factors, such as chronic inflammation, aging-related biological pathways.

## Conclusion

The prevalence of more than two chronic conditions was significantly higher in individuals with cognitive frailty, particularly those with SCD and SG. This indicates that the combination of cognitive decline and SG may be a critical marker for identifying individuals at risk of multimorbidity and serves as a critical marker for identifying high-risk groups, guiding middle-aged/older adults screening in communities and clinics. Additionally, osteoporosis, fractures, and the presence of more than two CCVD were more frequently observed in individuals with cognitive frailty. We recommend that walking speed and subjective cognition be closely monitored in adults over 50 years of age. Interventions aimed at strengthening physical exercise and improving walking speed may not only prevent AD but also mitigate chronic conditions.

## Data Availability

The original contributions presented in the study are included in the article/supplementary material, further inquiries can be directed to the corresponding author/s.
