# Correction: Multimorbidity patterns and cognitive frailty in adults aged over 50 years: China perspective

**DOI:** 10.3389/fpubh.2025.1758984

**Published:** 2026-01-12

**Authors:** Jiehua Zhu, Chenxi Ren, Tingjun Hu, Jun Jin, Qihao Guo

**Affiliations:** Department of Gerontology, Shanghai Sixth People's Hospital, Shanghai Jiao Tong University School of Medicine, Shanghai, China

**Keywords:** multimorbidity, slow gait, cognitive frailty, aged over 50 years, mild cognitive impairment

The citations for Lv et al. (2025) (37), Aprahamian et al. (2020) (38), and Grande et al. (2021) (39) were not correctly formatted in the article.

The citations have now been correctly formatted and inserted in the section **Discussion**, paragraphs 4 and 5 and added to the reference list.

The corrected paragraphs should read:

“The association between CCVD and SG, SCD, or cognitive frailty is well supported by prior research, but our study advances this understanding by focusing on their co-occurrence in SCD-SG, MCI-SG. Notably, while the rising prevalence and mortality of cardiovascular disease (CVD) in China underscore the clinical relevance of these links (29), prior work—including a recent study (37) has primarily focused on aggregate multimorbidity burden or isolated cognitive or gait outcomes rather than their combined presentation in preclinical stages.”

“Some studies reported that cardiovascular and metabolic multimorbidity correlates with incident cognitive frailty in older adults, emphasizing shared inflammatory pathways (38, 39). Our findings align with this mechanistic framework: elevated IL-6 and C-reactive protein have been linked to SG (30), reduced gray matter and hippocampal volume (31, 32)—structures critical for cognition and gait regulation—and subsequent cognitive decline (33). However, we extend this work by demonstrating that these inflammatory pathways also intersect with gender-specific comorbidities in preclinical AD subgroups, a gap not addressed in prior research.”

The figure citations incorrectly cited only the part figures instead of the whole figure. The citation “Figure 1A” should be edited to “Figure 1”; and the citation “Figure 2A” should be edited to “Figure 2”.

The original version of this article has been updated.
